# Commentary: A pointer about grasping numbers

**DOI:** 10.3389/fpsyg.2015.00227

**Published:** 2015-03-03

**Authors:** Martin H. Fischer, Elena Sixtus, Silke M. Göbel

**Affiliations:** ^1^Cognitive Sciences Division, University of PotsdamPotsdam, Germany; ^2^Department of Psychology, University of YorkYork, UK

**Keywords:** numerical cognition, embodied cognition, gestures, numeracy training, mathematical cognition

Mathematical skills are a key predictor of individual as well as economic success (Butterworth et al., 2011). Appropriate early training is therefore desirable, and recently, portable educational computer games for children have started to contribute to feeding this demand (e.g., Couse and Chen, 2010). The effectiveness of most such mobile training applications has not been properly evaluated. One key question is whether the limited manual interactions afforded by tablets and smartphones impoverish learning. From an embodied cognition perspective (e.g., Barsalou, 2008; Kontra et al., 2012; see also Montessori, 1948; Hebb, 1949) rich and varied sensory–motor activity supports learning while merely gesturing or swiping at everything prevents it (e.g., Spitzer, 2013; Tan et al., 2013). In sharp contrast, Novack et al. (2014) recently claimed that mere gesturing supersedes the value of physical object manipulation for mathematical learning.

Using magnetic number symbols on a whiteboard, Novack et al. (2014) studied how different object-directed hand actions influence children's learning of addition procedures. The authors found better knowledge generalization after gesturing to numbers compared to both direct and pantomimed number manipulations. They concluded that generalization of conceptual knowledge benefits from indirect object interactions (gesturing instead of manipulating). Instead, we argue here that poor performance in the direct manipulation condition reflects (1) interference with previous knowledge, (2) contra-productive attentional focusing, and (3) increased task complexity when compared with gesturing. The conclusions drawn by Novack et al. are thus not warranted and might mislead educational practitioners.

The children in Novack et al.'s study saw single equations of the form “2 + 9 + 4 = __ + 4” written on a whiteboard; all number symbols were covered with matching number magnets. They learned one of three strategies to equate both sides of such equations: (1) action: children in this group picked up those two magnets whose shapes differed from the right-side digit and moved them from the left side of the equation into the placeholder position on the right side; or (2) concrete gesture: these children pantomimed the action described in (1) without physically moving the magnets; or (3) abstract gesture: children in this group pointed with the fingers of one hand to the two digits on the left side and then to the placeholder. This is illustrated in our Figure 1.

**Figure 1:**
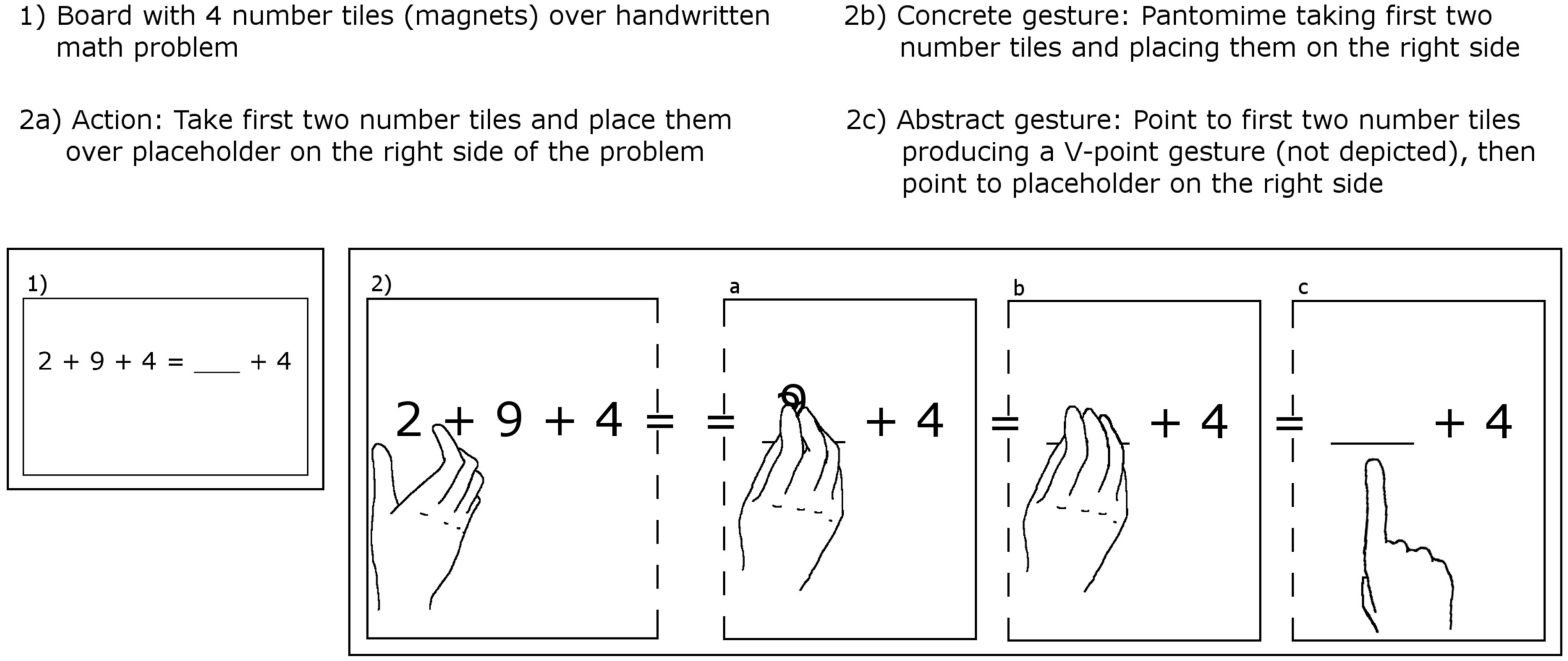
**Illustration of experimental conditions in Novack et al. (2014). Panel 1: Sample problem. Panel 2 a–c: right side of equation in the three experimental conditions**.

After solving six such problems by taking turns with the experimenter, all children were asked to first solve three more structurally identical problems, then three problems that required moving the second and third left-side addends (near transfer test) and finally two problems with no digits on one side repeating on the other side of the equation (far transfer test). The authors found that all trainings were equally effective for structurally identical problems but that a much smaller percentage of children from the action condition transferred their newly acquired knowledge to the novel problems, compared to abstract gesturing. However, their conclusion that gesturing is better suited for knowledge transfer than actual object manipulation is not warranted, for several reasons.

First, we note that the action condition required children to physically move two magnets from one side of the equal sign to the other, thus transforming the expression “2 + 9 + 4 = _ + 4” into “2 + 9 + 4 = 29 + 4” (see their Figure 1). Even if the two magnets are placed on top of each other, children will be left with factually wrong statements being considered as correct behavior. Thus, poor transfer following the action training can be explained as a trivial consequence of learning to generate false arithmetic statements, or at least of the added challenge to deal with the confusion generated by this procedure. Failing to generalize this “knowledge” merely indicates interference with previously learned arithmetic facts, a problem not present in the other two conditions.

Second, there was relatively good performance in the abstract gesture group; this probably reflects more effective (but contra-productive) visual filtering in the other two conditions, due to stronger engagement of object manipulation mechanisms. Object handling directs visual attention onto those objects and grasp preparation focuses attention to the object's size, thereby excluding other objects from processing (Schiegg et al., 2003; Fischer and Hoellen, 2004; Symes et al., 2008). Thus, reaching for and especially picking up two number magnets allowed the least processing of the other numbers present. This helps to explain why the action and concrete gesture groups both performed poor at far-transfer problems (see their Figure 3C) where different numbers appeared on the left and right sides of equations.

Third, control groups are needed to properly evaluate further possible contributors to the results, most important among them the cognitive load exerted by manual pre-shaping and alignment to an object (e.g., von Hofsten, 2007); this motoric load left the action task with the least remaining resources for learning relational problem features and for subsequent knowledge transfer. Moreover, gesture-unrelated cognition, such as passively observing the experimenter's differentially complex actions, also contributed to performance in all groups.

Further issues arise from reporting percentages instead of numbers of successful children in Figure 3. Re-converting Figure 3B, we found that in absolute numbers more, not fewer children benefited from pantomime than from gesturing (12 vs. 10), and we are left wondering why Figure 3C is confined to learners who solved both near- and far-transfer problems, although it is supposed to mirror generalization performance, while the so-called generalization test previously only contained far-transfer problems. It also remains unclear which training conditions are represented by those children who solved far-transfer but not near-transfer problems and/or trained problems and do therefore not appear in Figure 3C.

There is clear evidence that task-relevant whole body actions lead to better performance and more generalization in numeracy training (e.g., Fischer et al., 2011; Link et al., 2013) than just providing the answer by gesturing. We do therefore not debate the usefulness of gestures in learning arithmetic but we highlight that, due to the nature of the action condition in Novack et al., their results seriously underestimate the power of complex motor actions in training and teaching.

## Conflict of interest statement

The authors declare that the research was conducted in the absence of any commercial or financial relationships that could be construed as a potential conflict of interest.
